# Development of a caffeic acid–phthalimide hybrid compound for NADPH oxidase inhibition†

**DOI:** 10.1039/d1ra01066b

**Published:** 2021-05-18

**Authors:** Willian Henrique dos Santos, Maurício Ikeda Yoguim, Regina Gomes Daré, Luiz Carlos da Silva-Filho, Sueli Oliveira Silva Lautenschlager, Valdecir Farias Ximenes

**Affiliations:** Department of Chemistry, Faculty of Sciences, UNESP – São Paulo State University 17033-360 Bauru São Paulo Brazil valdecir.ximenes@unesp.br +55 14 3301 6088; Department of Pharmaceutical Sciences, Maringa State University (UEM) Maringa Paraná Brazil

## Abstract

NADPH oxidases are pharmacological targets for the treatment of inflammation-based diseases. This work presents the synthesis and study of a caffeic acid/phthalimide hybrid compound (C2) as a potential inhibitor of NADPH oxidases. Throughout the study, we have compared compound C2 with its precursor caffeic acid (C1). The redox properties were compared using three different antioxidant methodologies and showed that C2 was slightly less effective than C1, a well-established and robust antioxidant. However, C2 was three-fold more effective than albumin (used as a model protein). This chemical feature was decisive for the higher efficiency of C2 as an inhibitor of the release of superoxide anions by stimulated neutrophils and enzymatic activity of cell-free NADPH oxidase. Docking simulation studies were performed using the crystal structure of the recombinant dehydrogenase domain of the isoform NOX5 of *C. stagnale*, which retains the FAD cofactor (PDB: 5O0X). Considering that C2 could bind at the FAD redox site of NOX5, studies were conducted by comparing the interactions and binding energies of C1 and C2. The binding energies were −50.30 (C1) and −74.88 (C2) (kJ mol^−1^), which is in agreement with the higher efficacy of the latter as an NADPH oxidase inhibitor. In conclusion, incorporating the phthalimide moiety into caffeic acid was decisive for its effectiveness as an NADPH oxidase inhibitor.

## Abstract 2

The incorporation of the phthalimide moiety into caffeic acid was decisive for its effectiveness as an NADPH oxidase inhibitor.

## Graphical abstract

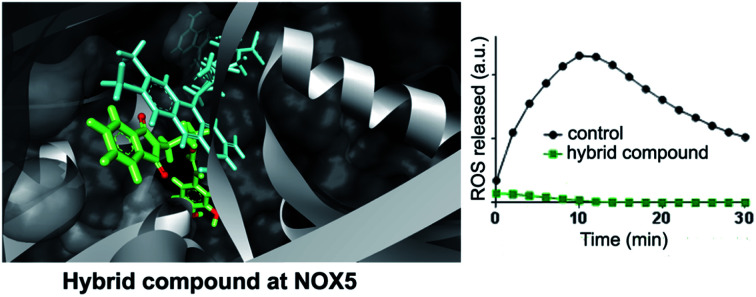

## 1. Introduction

NADPH oxidases (NOX) comprise a family of membrane proteins whose physiological function is to catalyze superoxide anion or hydrogen peroxide production. Indeed, they are the only known enzymes whose physiological role is to produce reactive oxygen species (ROS). The membrane catalytic subunits differentiate the seven isoforms, NOX1 to 5 and two dual oxidases DUOX1 and 2, associated with the membrane protein p22phox with cytosolic regulatory subunits; altogether, they form the NADPH oxidase complexes.^1–6^ NADPH oxidase substrates are molecular oxygen and the reduced coenzyme NADPH. The catalytic pathway involves electron transfer from cytosolic NADPH to the FAD prosthetic group in the dehydrogenase C-terminal cytosolic domain. Then, heme groups in the N-terminal transmembrane domain mediate the electron transfer to oxygen, leading to superoxide anion release in vacuoles or the extracellular medium.^7,8^ Superoxide anions and downstream ROS play fundamental roles in innate immunity^9,10^ and as signaling molecules involved in proliferation, apoptosis, differentiation, and migration.^11–13^ In phagocytes, the production of ROS is fundamental for the microbicidal capacity of these cells. Phagocytes express a large amount of NOX2, which is activated in response to external stimuli triggering a cascade of enzymatic reactions that convert superoxide anion to hydrogen peroxide and hypochlorous acid, which have potent microbicidal properties.^14–16^ Another example is endothelial cells, where NADPH oxidases (NOX1, 2, 4, and 5) are expressed in lower levels and distributed throughout subcellular compartments. In these cells, non-cytotoxic levels of ROS are related to redox signaling in normal cellular metabolism.^17,18^

Once unregulated ROS-mediated signaling is involved in many disease mechanisms, NADPH oxidases are attractive pharmacological targets for treating inflammatory-based diseases.^19,20^ The scientific literature is rich in studies where small molecules are proposed as NADPH oxidases inhibitors. For these compounds, the mechanisms of action are diverse. For instance, VAS2870, a triazole pyrimidine derivative, inhibits NOX2 by preventing the formation of the enzymatic complex.^21^ Apocynin, a controversial^22^ but still the most used NADPH oxidase inhibitor, acts by impeding the cytosolic oxidase components to translocate to the membrane.^23^ Phenyleneiodonium, even though potent, acts as a general flavoenzymes inhibitor.^24^

The phthalimide moiety is present in many molecules with potential pharmacological properties. The most famous example of a phthalimide-based drug is thalidomide.^25^ It has returned to the market and today is used to treat multiple myeloma,^26^ myelodysplastic syndrome,^27^ and autoimmune diseases.^28–30^ The phthalimide moiety's relevance in numerous hybrid compounds to improve their beneficial biological properties has been demonstrated. For instance, as an anti-inflammatory drug *via* cyclooxygenase-2 inhibition,^31^ lipoxygenase inhibition,^32^ inhibition of lipopolysaccharide-stimulated nitric oxide production in macrophage,^33^ prevention of vaso-occlusion and inflammation associated with the sickle cell anemia^34^ and impairment of TNF-alpha secretion and reduced IL-1 beta production.^35^

Caffeic acid and its derivatives also have numerous pharmacological effects. However, while phthalimide is synthetic, caffeic acid and its products are widely present as phytochemicals in plants. Among the natural derivatives of caffeic acid, it deserves particular attention caffeic acid phenethyl ester, a compound present in some propolis varieties. This compound has impressive health benefits, including antimicrobial, anti-inflammatory, antioxidant, anticancer, and immunomodulatory effects.^36–39^ Caffeic acid phenethyl ester is also able to inhibit NOX2, and this effect is related to its antioxidant activity and its lipophilic property.^40^ Based on the biological features of caffeic acid phenethyl ester and phthalimide, this work aimed to develop and study the capacity of a caffeic acid–phthalimide hybrid compound as an NADPH oxidase inhibitor.

## 2. Material and methods

### 2.1 Chemicals and solutions

Caffeic acid, *N*-(3-bromopropyl)phthalimide, human serum albumin (HSA) fatty acid and globulin free, 2,4,6-tri(2-pyridyl)-*s*-triazine (TPTZ), 2,2-diphenyl-1-picrylhydrazyl (DPPH), 2,2′-azobis(2-amidinopropane)hydrochloride (AAPH), 8-hydroxypyrene-1,3,6-trisulfonic acid trisodium salt (pyranine), luminol, HBSS (Hank's balanced salt solution), Histopaque®-1077 and -1119, lucigenin, NADPH (β-nicotinamide adenine dinucleotide 2′-phosphate reduced tetrasodium salt hydrate) and phorbol-12-myristate-13-acetate (PMA) were purchased from Sigma-Aldrich Chemical Co. (St. Louis, MO, USA). 2-(4-Iodophenyl)-3-(4-nitrophenyl)-5-(2,4-disulfophenyl)-2*H*-tetrazolium monosodium salt (WST-1) was purchased from Santa Cruz Biotechnology (Santa Cruz, CA, USA). Bradford reagent was obtained from Bio-Rad (California, USA). Stock solutions of the studied compounds (10 mmol L^−1^) were prepared in dimethyl sulfoxide. From the stock solutions, working solutions (1 mmol L^−1^ or less) were prepared in 50 mmol L^−1^ phosphate buffer at pH 7.0. HSA was dissolved in 50 mmol L^−1^ phosphate buffer at pH 7.0 to give a 1.0 mmol L^−1^ stock solution. PMA stock solutions were prepared in DMSO at a concentration of 100 μmol L^−1^. Ultrapure Milli-Q water from Millipore (Belford, MA, USA) was used to prepare buffers and solutions. All reagents were of analytical grade.

### 2.2 Synthesis and characterisation

The synthesis of (*E*)-3-(1,3-dioxoisoindolin-2-yl)propyl-3-(3,4-dihydroxyphenyl)acrylate (C2) was accomplished using a reported method with modifications.^41^ A mixture of caffeic acid (C1) (1.36 g, 7.57 mmol) and NaHCO_3_ (0.636 g, 7.57 mmol) was added to dimethylformamide (20 mL) and stirred at 60 °C for two hours under nitrogen, then at 100 °C for 15 min. Freshly distilled and acid-free *N*-(3-bromopropyl)phthalimide (2.44 g, 9.08 mmol) was added, and the mixture stirred for an additional 1.25 h at 100 °C. The mixture was poured into dilute aqueous HCl and extracted with diethyl ether. The ether layer was washed several times with water, then with saturated brine, and dried with MgSO_4_. The solvent was removed under vacuum to give the pure C2 as an off-white solid (1.72 g, 62%). ^1^H NMR (DMSO-d_6_, 400 MHz): *δ* (ppm) 9.61 (s, 1H), 9.15 (s, 1H), 7.85 (m, 1H), 7.79 (m, 2H), 7.41 (m, 1H), 6.94 (m, 1H), 6.88 (dd, *J* = 8.3, 2.0 Hz, 1H), 6.74 (d, *J* = 8.3 Hz, 1H), 6.08 (m, 1H), 4.13 (t, *J* = 6.1 (×2) Hz, 2H), 3.71 (t, *J* = 6.1 (×2) Hz, 2H), 1.98 (t, *J* = 6.1 (×2) Hz, 2H). ^13^C NMR (DMSO-d_6_, 100 MHz): *δ* (ppm) 167.9, 166.3, 148.4, 145.5, 145.0, 134.3, 131.7, 125.4, 122.9, 121.2, 115.7, 114.8, 113.6, 61.9, 34.9, 27.0. ESI-MS: calculated [M − H]^−^: 366.10; found 366.13 (see ESI, Fig. S1 and S2†).

### 2.3 Isolation of human neutrophils

Venous blood was collected from healthy volunteers. Neutrophils (polymorphonuclear cells, PMN) were separated by centrifugation on a Histopaque®-1077/1119 gradient at 700 g for 30 min at room temperature.^42^ Subsequently, the cells were resuspended in fresh HBSS (140 mM NaCl, 5 mM KCl, 20 mM HEPES, 4 mM glucose, 2 mM CaCl_2_, pH 7.4). All experiments were performed in accordance with the Guidelines of the Research Ethics Committee of the School of Pharmaceutical Sciences and approved by the ethics committee at Sao Paulo State University. Informed consents were obtained from human participants of this study.

### 2.4 Total ROS production assay

PMN cells (1 × 10^6^ cells per mL) in the absence (control) and presence of the studied compounds were pre-incubated in HBSS at 37 °C for 10 min using a flat bottom white microplate. Then, luminol (10 μmol L^−1^) was added, and the reaction was triggered by adding PMA (0.1 μmol L^−1^). The light emission was measured for 30 min at 37 °C using a plate luminometer (Centro Microplate Luminometer LB960, Berthold Technologies, Oak Ridge, TN, USA). The final reaction volume was 250 μL. The total ROS production was evaluated by luminol-enhanced chemiluminescence.^43^ The integrated light emission was used as an analytical parameter for the measurement of total ROS production. The inhibitory potency was calculated using the light emission generated by the positive control (100%) as reference.

### 2.5 Superoxide anion radical assay

PMN cells (1 × 10^6^ cells per mL) in the absence (control) and presence of the studied compounds were pre-incubated in HBSS at 37 °C for 10 min using a flat bottom transparent microplate. Then, WST-1 (500 μmol L^−1^) was added, and the reaction was triggered by adding PMA (0.1 μmol L^−1^). The reaction mixtures were incubated for 30 min at 37 °C. The extracellular release of superoxide anion radical was measured by the reduction of WST-1.^44^ The absorbance increase at 450 nm was measured using a Spectramax M2 microplate reader (Molecular Devices, Sunnyvale, CA). The inhibitory potency was calculated based on the absorbance of positive control, in which the PMN cells were stimulated and incubated in the absence of the studied compounds.

### 2.6 NADPH oxidase enzymatic activity assay

PMN cells (1 × 10^6^ cells per mL) were treated with the studied compounds (10 and 100 μmol L^−1^) for one hour at 37 °C under agitation. Then, the cells were exposed to PMA (0.1 μmol L^−1^) for 30 min at 37 °C, followed by sonication (4C15, Branson Ultrasonics) on ice for 120 s (40% amplitude, 5 s on and 15 s off). The cell lysates were incubated with 50 μmol L^−1^ of lucigenin for 5 min, followed by incubation with 200 μmol L^−1^ of NADPH for 20 min.^45,46^ The production of superoxide anion was immediately measured through lucigenin-enhanced chemiluminescence. The readings were performed in a luminometer (SpectraMax® L, Molecular Devices) and the results normalized by protein concentration (Bradford reagent) at 595 nm (Bio-Tek®, Power Wave XS), using bovine serum albumin as a standard for the calibration curve. NADPH oxidase activity was expressed as relative luminescence unit per μg protein.

### 2.7 Protein binding assays

HSA (5.0 μmol L^−1^) was titrated with the studied compounds (0–14 μmol L^−1^) in 50 mmol L^−1^ phosphate buffer, pH 7.0, 25 °C. After each addition, the protein/ligand mixtures were incubated for 2 min before the fluorescence measurements.^47^ The fluorescent intensities were corrected for the inner filter effect caused by attenuation of the excitation and emission signals provoked by the UV-Vis absorption using eqn (1). In this equation, *F*_corr_ and *F*_obs_ are corrected and observed fluorescence intensities, respectively. Ab_ex_ and Ab_em_ are the absorptions at 295 nm and 343 nm, the wavelengths of excitation and emission.1
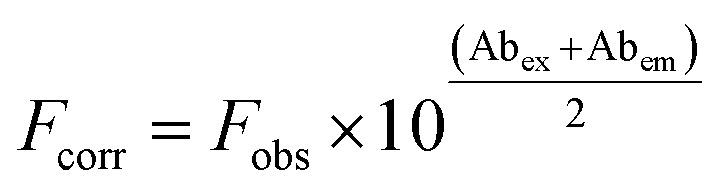


The Stern–Volmer (*K*_sv_) and bimolecular quenching (*k*_q_) constants were obtained by fitting the experimental data to eqn (2). In this equation, *F*_0_ and *F* are the fluorescence intensity in the quencher's absence and presence, Q is the quencher (studied compounds) concentration, *k*_q_ is the bimolecular quenching constant, *τ*_0_ is the average lifetime of the fluorophore tryptophan in BSA.^48^2
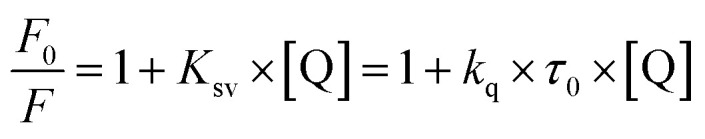


The association constants were obtained by nonlinear fitting of eqn (3). In this equation, *F*_0_ is the fluorescence in the absence of quencher, *F* is the fluorescence in the presence of quencher, *φ* is the fluorescence ratio change amplitude, *P* is the protein concentration, Q is the concentration of added quencher, and *K*_d_ is the dissociation constant.^48^3
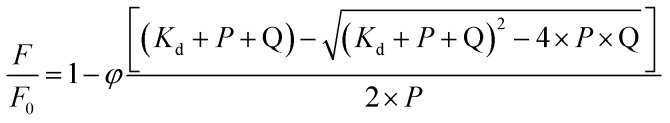


The experiments were performed using a 3 mL quartz cuvette with a 10 mm path length and magnetically stirred during the measurements. The absorbance and fluorescence spectra were measured using a Perkin Elmer Lambda 35 UV-visible spectrophotometer and Perkin Elmer LS 55 spectrofluorimeter, respectively (Shelton, CT, USA). The linear and nonlinear fittings for Stern–Volmer and association constants were obtained using the GraphPad Prism version 5.00 for Windows (GraphPad Software, San Diego, California USA).

### 2.8 Circular dichroism assay

The electronic circular dichroism spectra of the studied compounds (30.0 μmol L^−1^) were recorded in the absence and presence of HSA (30.0 μmol L^−1^). The baseline (50 mmol L^−1^ phosphate buffer, pH 7.0) was subtracted from all the measurements. The complexation was conducted at 25 °C. The electronic circular dichroism spectra were recorded in a Jasco J-815 spectropolarimeter (Jasco, Japan) equipped with a thermostatically controlled cell holder. The spectra were obtained with 1 nm step resolution, the response time of 1 s, and scanning speed of 100 nm min^−1^. A 3 mL quartz cuvette with a 10 mm path length and a magnetic stirrer was used for the measurements in the near-UV-CD range.^49^

### 2.9 DPPH scavenging assay

The studied compounds were incubated for 30 min with 100 μmol L^−1^ DPPH dissolved in ethyl alcohol in the dark. The scavenging activity was evaluated spectrophotometrically at 517 nm using the unreacted DPPH radical absorbance as control and calculated as follows: [(absorbance of control − absorbance of sample)/(absorbance of control)] × 100.^50^

### 2.10 Ferric reducing antioxidant power (FRAP) assay

The FRAP reagent was prepared as follows: 1 mL TPTZ (10 mmol L^−1^ dissolved in 40 mmol L^−1^ HCl), 1 mL FeCl_3_ (20 mmol L^−1^ dissolved in water), and 10 mL sodium acetate buffer, 300 mmol L^−1^, pH 3.6. The fixed volume (10 μL) of the studied compounds was incubated with 290 μL of FRAP reagent for 30 minutes in the dark at room temperature at increasing concentrations. The absorbance was measured at 593 nm using the FRAP reagent as blank. The relative antioxidant efficacy was evaluated by the slope of the analytical curves.^50^

### 2.11 Peroxyl radical scavenging assay

Pyranine (10 μmol L^−1^) was incubated with 20 mmol L^−1^ AAPH in 50 mmol L^−1^ phosphate buffer, pH 7.0 at 37 °C in the absence (control) or presence of the studied compounds using a flat bottom black microplate. The reactions were conducted for two hours, and the pyranine fluorescence bleaching was monitored at 460/510 nm using a Spectramax M2 microplate reader (Molecular Devices, Sunnyvale, CA). The final reaction volume was 250 μL. The area under the curve (AUC) of fluorescence *versus* time graphs, in the absence and presence of the studied compounds, was plotted against concentration.^51^ Analytical curves obtained by plotting AUC_s_/AUC_c_ (sample/control) against concentrations were built. The slopes were used as the analytical parameter.

### 2.12 Molecular docking simulation

The crystallographic structure of the catalytic flavin adenine dinucleotide (FAD) dehydrogenase domain of *C. stagnale* NOX5, csDH domain (PDB ID code 5O0X, resolution: 2.2 Å), was used in the modeling studies.^52^ Simulations were carried out using GOLD 5.2 (Genetic Optimization for Ligand Docking), a software-based on a genetic algorithm to explore the ligand conformational space and fitness function GoldScore. The simulations provided the best ten structures for the binding site. The GOLD criterium consisted of choosing the best structures out of the ten available provided by the program. The preferred structure must have overlap and score value closest to the total average.^53^ The protein was prepared for docking studies by adding hydrogen atoms, removing water, and co-crystallized inhibitors. The protein-ligand interactions and images were obtained using the software Discovery Studio Visualizer 2017 R2 Client.

### 2.13 Statistics

The results were expressed as mean ± SD. All tests were performed in triplicates. Statistically significant differences were determined by the One-Way ANOVA and Turkey's multiple comparison test.

## 3. Results and discussion

### 3.1 Design and redox properties

**Fig. 1:**
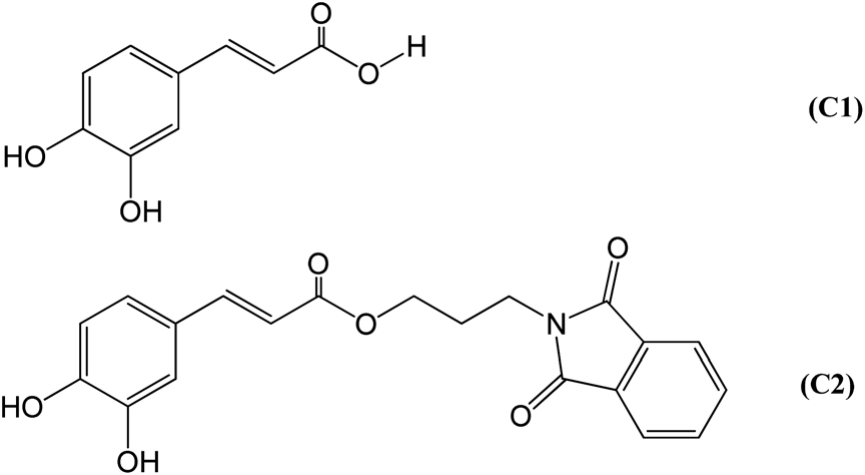
Molecular structures of the studied compounds.

The design of the hybrid caffeic acid/phthalimide compound (C2) (Fig. 1) was based on the previously described pharmacological properties of its precursors^33–39,54–56^ and the beneficial effect of an increased hydrophobicity for an NADPH oxidase inhibitor.^40,57,58^C2 was synthesised through a one-step reaction between caffeic acid (C1) and *N*-(3-bromopropyl)phthalimide, two commercial and relatively inexpensive chemicals. The higher hydrophobicity index of C2 (log *P* = 2.27) was obtained by the ester moiety and the aliphatic three-carbon chain linkage between C1 (log *P* = 1.15) and the phthalimide nucleus.

NADPH oxidases' physiological role is to catalyse the reduction of molecular oxygen to superoxide anion or hydrogen peroxide.^14–16,59^ Once the NADPH oxidase enzymatic activity is determined by measuring superoxide anion production, the enzymatic complex's inhibitors are potentially indistinct from ROS scavengers. This consideration is particularly relevant when the studied inhibitor has antioxidant features. It is the case of C1 and C2. Hence, to discriminate direct ROS scavenging activity from inhibition of NADPH oxidase enzymatic activity, C2 was initially compared with C1, a well-established antioxidant,^60^ as ROS scavengers.

**Fig. 2:**
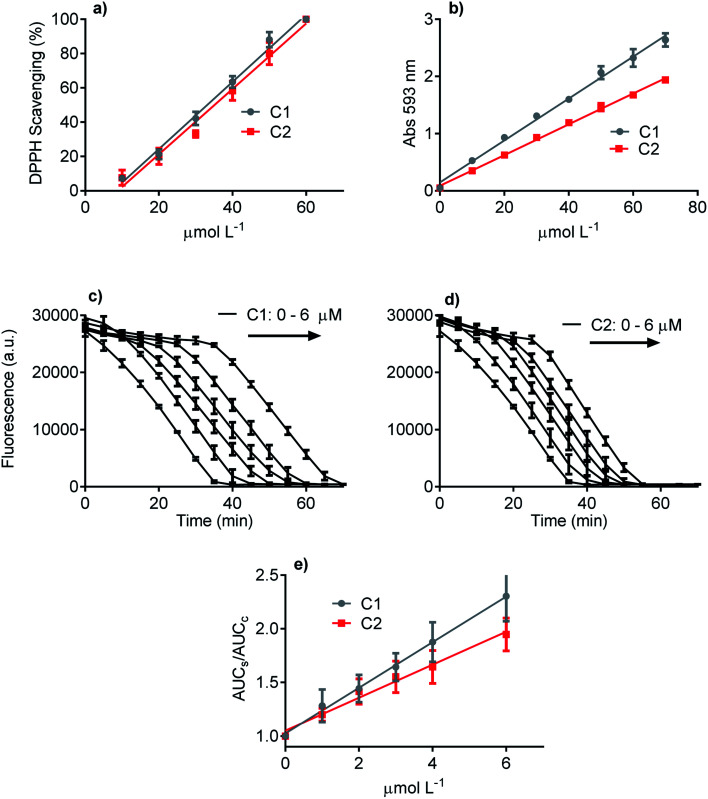
The relative antioxidant potency of C1 and C2. (a) Scavenging of DPPH free radical. The EC_50_ were 33.0, 38.1 for C1 and C2, respectively (*R*^2^ > 0.9523). (b) Reduction of ferric ion (FRAP assay). The slopes were 0.039 and 0.028 *Δ*_abs_/μmol L^−1^ for C1 and C2, respectively (*R*^2^ > 0.9873). (c–e) Scavenging of peroxyl radical (pyranine assay). The slopes were 0.212 and 0.157 ΔAUC_s–c_/μmol L^−1^ for C1 and C2, respectively (*R*^2^ > 0.9102).

Three methodologies were used to evaluate and compare the redox properties of the studied compounds. The first one was the widely used DPPH scavenging assay, which is based on the reduction of the stable DPPH radical.^50^Fig. 2a shows that C1 and C2 were efficient and statistically similar as electron donors to the DPPH free radical. The efficacy of both compounds as antioxidants can be attested by the EC_50_ values (C1 33 μmol L^−1^ and C2 38 μmol L^−1^), which are close to those usually reported for potent antioxidants as ascorbic acid, trolox, and rosmarinic acid.^58,61^ The second antioxidant methodology was the FRAP assay (Fig. 2b). This analytical technique is based on the efficacy as a reducing agent of ferric ions.^50^ How it can be seen, even though C1 was more potent than C2, both compounds can be regarded as efficient antioxidants. The slopes of the linear regression curves were 0.039 and 0.028 *Δ*_abs_/μmol L^−1^, respectively. Finally, the last applied antioxidant assay was based on peroxyl radicals scavenging.^51^ Again, C1 and C2 were effective competitors of pyranine by peroxyl radicals, which is indicative of the capacity of both compounds to react with this ROS.^62^ This result can be visualized by the concentration-dependent delay in pyranine fluorescence bleaching (Fig. 2c and d). In agreement with the FRAP assay, C1 was more effective as a peroxyl radicals scavenger than C2. In short, it can be concluded that the presence of the phthalimide moiety slightly decreased the ROS scavenging capacity of C1, a well-accepted and potent antioxidant. More importantly, in the context of this work, any improved effect of C2 as an inhibitor of NADPH oxidase in cell and cell-free enzymatic assays cannot be explained by direct ROS scavenger activity.

### 3.2 Interaction with protein

**Fig. 3:**
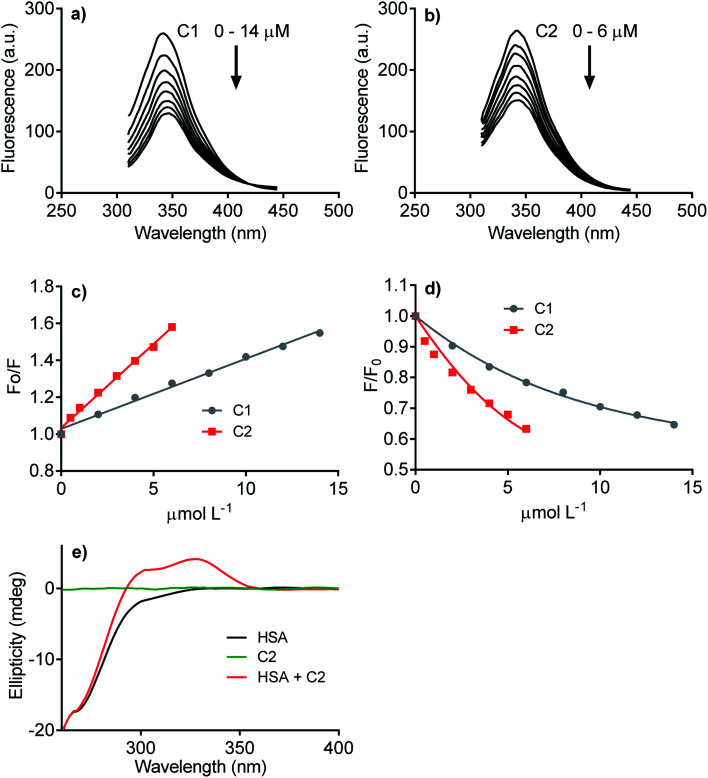
Interaction of the studied compounds with human serum albumin. (a, b) Quenching of intrinsic fluorescence of HSA. (c) Stern–Volmer plots (C1, *K*_sv_ = 3.8 × 10^4^ mol L^−1^; C2, *K*_sv_ = 9.1 × 10^4^ mol L^−1^), (d) association constant plots (C1, *K*_a_ = 1.5 × 10^5^ mol L^−1^; C2, *K*_a_ = 6.0 × 10^5^ mol L^−1^). (e) Circular dichroism spectrum of C2 in the absence and presence of HSA.

The next step was to evaluate how hydrophobicity's alteration could affect the hybrid molecule's capacity to interact with proteins. The experimental approach evaluated the affinity of C1 and C2 with human serum albumin (HSA), used as a model protein. As well-known, drug transportation is one of the physiological functions of this blood protein. This phenomenon is particularly relevant to hydrophobic endogenous and exogenous compounds as fatty acids and pharmaceutical drugs.^63^ The binding affinity of C1 and C2 was evaluated by HSA intrinsic fluorescence quenching. Fig. 3 shows the HSA-fluorescence quenching and highlights the higher efficacy of C2 compared to C1. Quantitatively, the interactions were assessed by the Stern–Volmer (*K*_sv_) and association (*K*_a_) constants. The *K*_sv_ constants revealed that the interaction of C2 (9.1 × 10^4^ mol L^−1^) was almost three-fold higher compared to C1 (3.8 × 10^4^ mol L^−1^) (Fig. 3a–c). The magnitudes of *K*_a_ were compatible with values usually observed for well-established ligands of HSA as naproxen^64^ and phenylbutazone,^65^ and C2 (6.0 × 10^5^ mol L^−1^) was about three-fold more effective than C1 (1.6 × 10^5^ mol L^−1^) (Fig. 3d). The interaction was also evaluated by induced circular dichroism (ICD). Fig. 3e shows that C2 is devoid of circular dichroism signal in aqueous solution; however, in the presence of HSA, a clear band was observed in a spectral region where the protein is transparent. This result is an example of induction of chirality in a ligand due to its fixation in a chiral microenvironment, *i.e.*, the protein cavities.^49,66^ In resume, these findings are consistent with our proposal, *i.e.*, the higher hydrophobicity of the hybrid molecule increased its affinity with protein. This chemical property can be beneficial regarding interaction with NOX. Obviously, HSA is just a model protein, and future studies must be performed with purified or recombinant NOX.

### 3.3 Inhibition of total ROS produced by neutrophils

**Fig. 4:**
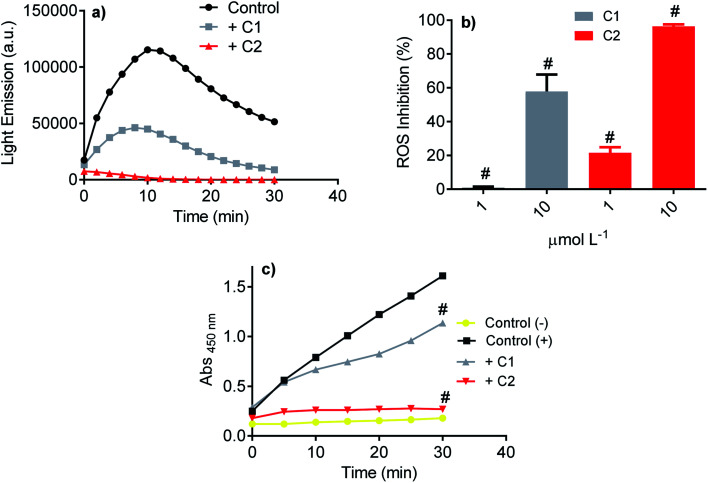
Inhibition of total ROS and superoxide anion produced by stimulated neutrophils. (a) Chemiluminescence emitted by activated neutrophils and inhibitory effect of studied compounds (10 μmol L^−1^). (b) Concentration-dependent inhibition of ROS (chemiluminescence assay). (c) Production of superoxide anion by activated neutrophils (positive control) and the inhibitory effect of the studied compounds (WST-1 assay). The results are mean and SD of triplicates. ^#^*p* < 0.01 compared with positive control.

Initially, the studied compound's efficacy as an inhibitor of NADPH oxidase was evaluated using the luminol-dependent chemiluminescence assay. It is a non-specific assay used to measure total ROS produced by activated leukocytes.^43^ Neutrophils are the most well-established source of NADPH oxidase, specifically NOX2. This isoform of NOX was the first characterized and its physiological function elucidated, *i.e.*, ROS generation for bacterial killing during the phagocytic process.^67–69^ In neutrophils, the superoxide anion is enzymatically or non-enzymatically dismutated to hydrogen peroxide. The last is the substrate of myeloperoxidase, which catalyzes chloride anion's oxidation to hypochlorous acid.^70^ All these ROS can oxidize luminol; hence, the chemiluminescence intensity is related to the activated cells' total ROS. Here, PMA acting as a soluble stimulus was used to activate NADPH oxidase in neutrophils. Fig. 4a shows the light emission profile elicited by PMA-activated neutrophils and the studied compounds' inhibitory effect. How it can be seen, both compounds were potent inhibitors, but C2 was significantly more efficient (Fig. 4b). The luminol-dependent chemiluminescence assay provided our first evidence that an improvement was obtained with the hybrid molecule. As the luminol assay detects total ROS, one cannot discard that the inhibition could result from ROS's direct scavenging effect. However, if so, C1 should be as effective or better than C2, since the first one was more potent ROS scavenger as demonstrated in the Section 3.1.

### 3.4 Inhibition of superoxide anion produced by neutrophils

A step further, the compounds were evaluated as specific inhibitors of the release of superoxide anion. NADPH oxidase activation in neutrophils was measured using the sulphonated tetrazolium salt (WST-1), a specific chromogenic probe to superoxide anion. WST-1 is water-soluble and membrane impermeable. Hence, the formazan salt produced by its reaction with superoxide anion can be detected in the extracellular medium using conventional absorbance measurements.^44^Fig. 4c shows the release of superoxide anion (positive control) compared to unstimulated cells (negative control). How it can be seen, C2 was again the more potent inhibitor, revealing its capacity as NADPH oxidase inhibitor and not only as a scavenger of ROS. A comparison can be made with apocynin, the phytochemical most used as NADPH oxidase.^71^ In the same experimental methodology (WST-1) and with the same number of cells, 10 μmol L^−1^ of apocynin inhibited only 5% of superoxide anion release^72^ while C2 reached 90%.

### 3.5 Inhibition of NADPH oxidase in the cell lysate

**Fig. 5:**
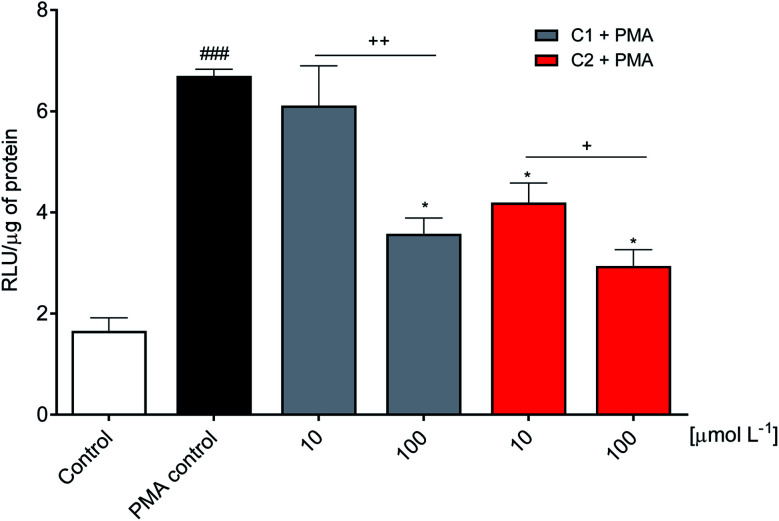
Inhibition of NADPH oxidase enzymatic activity. Relative concentration of superoxide anion production triggered by adding the coenzyme NADPH in neutrophil lysate. RLU: relative luminescence unit. Control: PMA non-activated and untreated cells. PMA control: PMA activated and untreated cells. ^###^*p* < 0.0001 compared with control, *p < 0.0001 compared with PMA control, ^+^*p* < 0.001, ^++^*p* < 0.0001.

The inhibitory effect observed using viable neutrophils was confirmed using a cellular lysate. This is an important issue since the NADPH oxidase complex's activation involves assembling cytosolic and membrane proteins. Hence, an inhibitor could act by different mechanisms, including the inhibition of cytosolic proteins' migration to the membrane.^73,74^ In the cell lysate assay performed here, the neutrophils were submitted to sonication to disrupt the cell membrane. Then, the substrate NADPH was added, and the enzymatic activity was measured. Fig. 5 shows that C2 was again the best inhibitor.

### 3.6 Molecular docking simulation

NADPH oxidases are multiprotein membrane complexes activated and regulated through the assembling of cytosolic protein components. The mechanisms of inhibition are diverse, including the impairment of migration of cytosolic oxidase components p47-phox and p67-phox to the membrane;^23,74,75^ preventing the formation of enzymatic complex;^21^ acting as general flavoenzymes inhibitor,^24^*etc.* For these reasons, *i.e.*, the absence of a unique mechanism of inhibition and the difficulty of obtaining the crystal structure of NOX, molecular docking simulations of potential inhibitor is still not typical in the scientific literature. In this context, recently, Magnani and collaborators deposited the first crystal structure of an NADPH oxidase (NOX5 isoform), obtained from a mutant of the recombinant protein obtained from *C. stagnale*, that retain the FAD cofactor in the crystal. Specifically, the dehydrogenase domain of *C. stagnale* (csDH, PDB access code 5O0X).^52^ The structure of the isolated NADP-binding lobe of human NOX2 is also available (PDB: 3A1F); however, the FAD-binding domain in this human NOX2 structure is not present. As Magnani and co-authors described, the thermal stability and retention of FAD in NOX5 were obtained by adding the amino acid sequence PWLELAAA after the C-terminal. This mutant enzyme retained the enzymatic activity. The docking studies showed that upon displacement of this amino acid sequence, NADPH was easily modeled to fit in the crevice at the FAD-binding interface.^52^

**Fig. 6:**
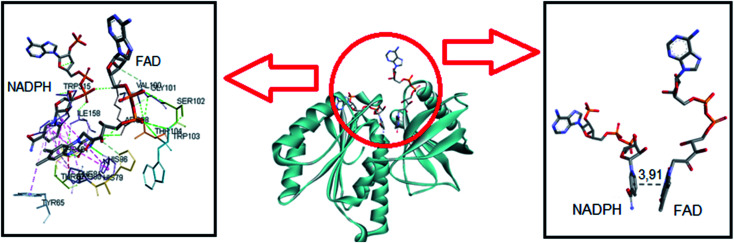
Representation of NADPH pose, interactions with amino acid residues, and FAD distance in the csDH domain of NOX5.

This was our starting point in the present study. We asked and kindly received from Dr Francesca Magnani from the University of Pavia, Italy, the PDB file of the csDH domain complexed with NADPH. Considering that C1 and C2 could bind at the FAD redox site, the studies were conducted by comparing the interactions and binding energies of C1 and C2 with those observed for NADPH. To do so, NADPH was removed from the crystal structure of the csDH domain and redocked using GOLD 5.5 software, as described in Section 2.12. NADPH's lower energy pose presented binding energy of −79.86 kJ mol^−1^ and π–σ interactions with Arg98, Val100, Arg264, Pro290, and leu293; and hydrogen bonds with Arg98, Gly157, Ile158, Arg193, and Arg264. The distance between the isoalloxazine ring of FAD and NADPH's nicotinamide ring was 3.91 Å (Fig. 6).

**Fig. 7:**
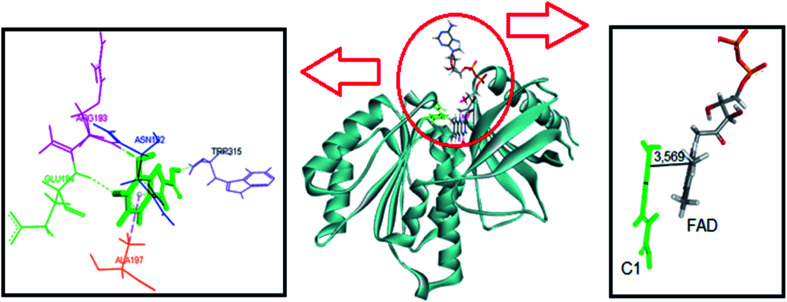
Representation of C1 pose, interactions with amino acid residues, and FAD distance in the csDH domain of NOX5.

**Fig. 8:**
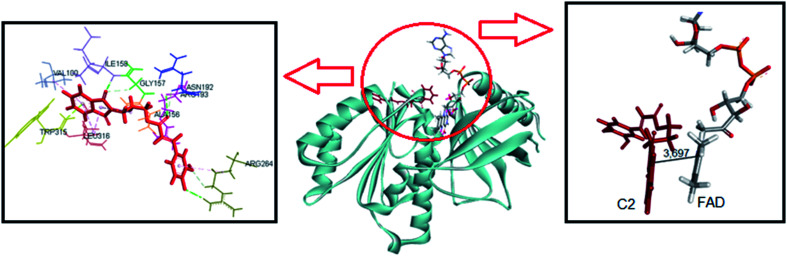
Representation of C2 pose, interactions with amino acid residues, and FAD distance in the csDH domain of NOX5.

The same computational approach was used to simulate C1 and C2 interactions with the csDH domain of NOX5. The reference was the binding site of NADPH and a cavity ratio of 7 Å, which provided the best results, *i.e.*, the lower energy difference between 10 poses. The C1 lower energy pose presented binding energy of −38.68 kJ mol^−1^ and π–σ interactions with Ala197 and hydrogen bonding with Asn192, Arg193, Glu194, Trp315 (Fig. 7). The distance between the isoalloxazine ring of FAD and the catechol moiety of C1 was 3.569 Å. This distance is similar to the distance between FAD and NADPH (3.91 Å). C2 showed binding energy of −57.25 kJ mol^−1^ and interactions π–σ with Val100, Ala156, Gly157, Asn192, Arg193, Arg264, Leu316, π–π interactions with Ala156 and Trp315, and hydrogen bonding with Ile158 and Arg264 (Fig. 8). The distance between the isoalloxazine ring of FAD and the phthalimide moiety of C2 was 3.697 Å. An improvement in the binding energies was obtained by flexibilization of the lateral chains in the amino acid residues of the csDH domain of NOX5. In this case, the values were changed to −50.30 and −74.88 kJ mol^−1^ for C1 and C2, respectively.

Table 1 resumes the binding energies and interactions. In short, the molecular docking simulations were consistent with the experimental results since C2 was the most effective ligand and could potentially compete with NADPH by the binding site in the csDH domain of NOX5. In short, compared to C1, the interaction of C2 with the csDH domain of NOX5 was significantly distinct. Besides the different amino acid interactions, the ring of C2 staking on isoalloxazine FAD is the phthalimide one, which is absent in C1. We believe this interaction must be involved in the higher efficacy of C2 as a NOX inhibitor. Obviously, the isoform tested experimentally was NOX2. However, these protein isoforms' presented high homology. Fig. S3† shows the superposition of NOX2 and NOX5 structures. In short, this computational study provides a putative molecular explanation for the higher affinity of C2 as an inhibitor.

**Table 1:** Binding energy and interactions of NADPH, C1, and C2 in csDH domain of NOX5

	NADPH	C1	C2
Energy (kJ mol^−1^)	−79.86	−50.30	−74.88
O–H	5	4	2
	Arg98, Gly157, Ile158, Arg193 and Arg264	Asn192, Arg193, Glu194 and trp315	Asn192, Arg193, Glu194 and trp315
π–σ	5	1	7
	Arg98, Val100, Arg264, pro290 and leu293	Ala197	Val100, Ala156, Asn192, Arg193, Arg264 and Leu316
π–π	0	0	2
			Ala156 and trp315

## 4. Concluding remarks

Phenolic acids and their derivatives can act as a scavenger (antioxidant effect), or source (pro-oxidant effect), or by inhibiting activity and expression of enzymes involved in the generation or degradation of ROS. In this way, phenolic acid's action mechanisms are linked to the modulation of ROS-dependent cellular signaling pathways of inflammatory and degenerative disorders.^76–81^ These mechanisms are diverse, and often difficult to distinguish whether these compounds act by ROS scavenging or inhibiting enzymes involved in their metabolism. This statement is particularly true for NADPH oxidases, which rely on the determination of superoxide anion to access the inhibitory effects of studied compounds. Hence, compounds with antioxidant features are usually regarded as ROS scavengers, and their real impact as NADPH oxidase activity is questioned.^22,82–85^ We propose that this is not always the case. In the last years, we have shown the efficacy of some phenolic acid derivatives as inhibitors of NOX2 and called the attention of readers for a real inhibition and not only scavenger effects.^57,58,72,86^ Here, we advanced in this proposal by developing the hybrid caffeic acid–phthalimide compound. As we have demonstrated, the hybrid compound's redox properties were not improved compared to caffeic acid, a widely recognized and potent ROS scavenger;^60^ actually, a slight decrease was observed. On the other hand, the capacity as a NOX2 inhibitor was significantly increased not only in cell-based assay but also using cell lysate. Hence, there is no doubt that an inhibitory effect on NOX2 enzymatic activity took place. Our last publications have argued that a unifying characteristic of the phenolic acid derivatives that presented inhibitory effect was their increased hydrophobicity.^57,58,72,86^ We propose that these redox-active lipophilic molecules could more easily access NADPH oxidases as a membrane protein. This property is also present in the hybrid molecule developed here. The higher lipophilicity of C2 also promoted increased binding energy with a model protein, suggesting that interaction with NOX's dehydrogenase domain could also be facilitated. Besides these chemical features, the phthalimide moiety in C2 increased the binding energy in the dehydrogenase domain of NOX5. In conclusion, besides antioxidant capacity and increased hydrophobicity, the phthalimide moiety was fundamental for C2 as a NOX2 inhibitor. Finally, even though C2 was an effective NOX2 inhibitor and the *in silico* experiments suggested that it could bind in the catalytic site of the enzyme complex, we cannot exclude other mechanisms of action, for instance, by blocking the assembling of the enzymatic complex.

## Author contributions

Conceptualization: VFX, WHS; funding acquisition: VFX, LCSF, SOSL; investigation: WHS, VFX, RGD, MIY; methodology: VFX, WHS, RGD, LCSF, SOSL; writing – original draft: VFX, WHS; writing – review & editing: VFX.

## Conflicts of interest

The authors have no conflicts of interest to declare.

## Supplementary Material

RA-011-D1RA01066B-s001
