# Supplementary material for: Development of a caffeic acid–phthalimide hybrid compound for NADPH oxidase inhibition
Source: RSC Adv. 2021 May 18;11(29):17880–90. doi: 10.1039/d1ra01066b (PMC9033209; doi:10.1039/d1ra01066b)
Supplement: RA-011-D1RA01066B-s001 [file RA-011-D1RA01066B-s001.pdf]

Electronic Supplementary Material (ESI) for RSC Advances.
This journal is © The Royal Society of Chemistry 2021

Electronic Supplementary Information

Development of a caffeic acid-phthalimide hybrid compound for NADPH oxidase inhibition

Willian Henrique dos Santos[1], Maurício Yoguim[1], Regina Gomes Daré[2], Luiz Carlos da Silva-Filho[1], Sueli Oliveira Silva Lautenschlager[2] and Valdecir Farias Ximenes[1,*]

[1]Department of Chemistry, Faculty of Sciences, UNESP - São Paulo State University, 17033-360, Bauru, São Paulo, Brazil. [2]Department of Pharmaceutical Sciences, Maringa State University (UEM), Maringa, Paraná, Brazil.

*Correspondence should be addressed to Valdecir Farias Ximenes.

Tel.: +55 14 3301-6088

http://orcid.org/0000-0003-2636-3080

Email: valdecir.ximenes@unesp.br

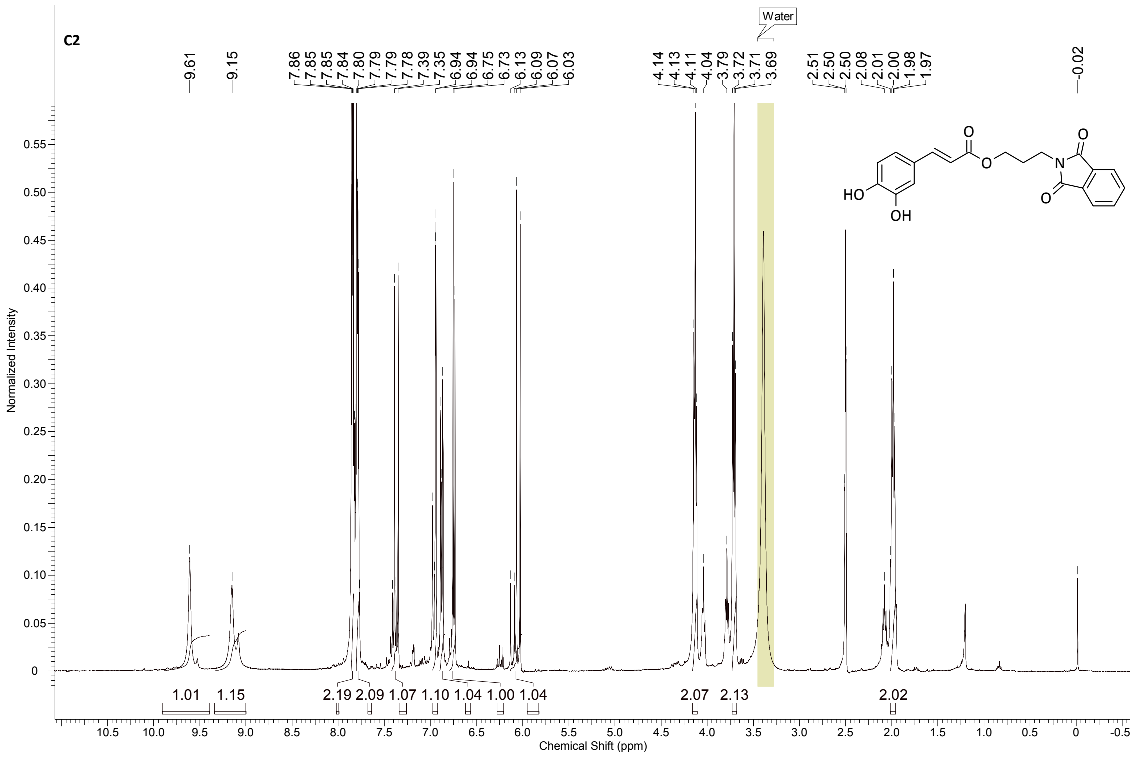

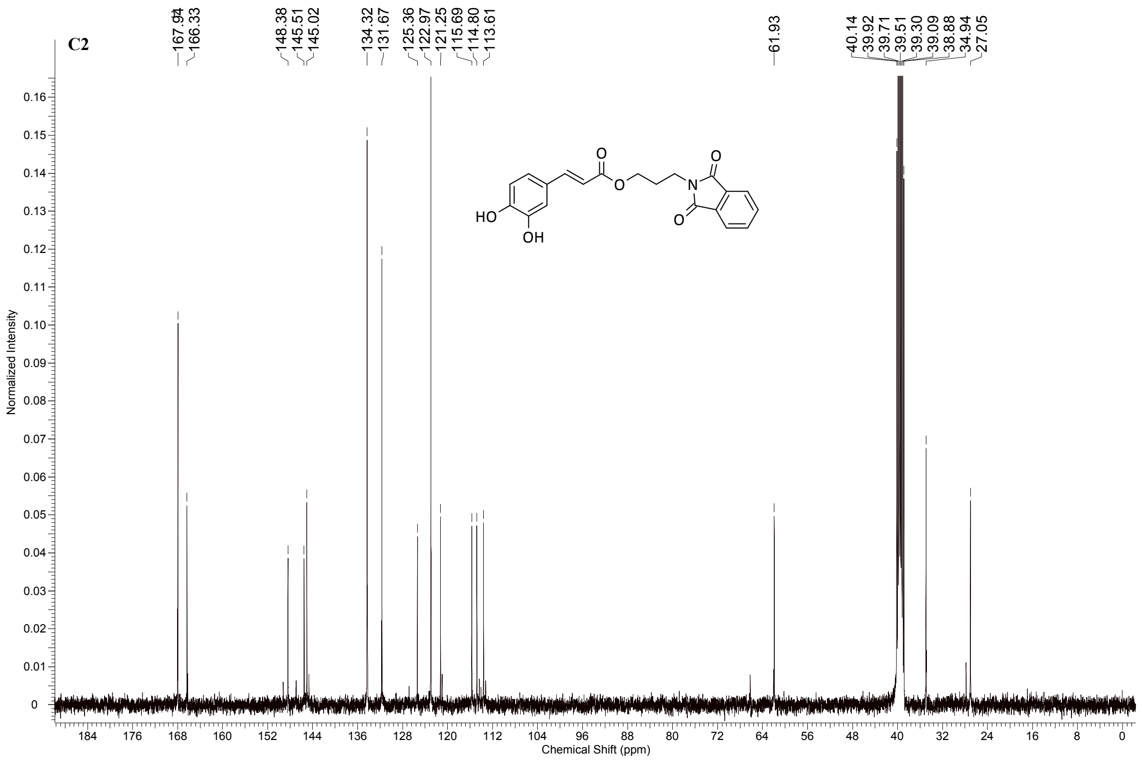

Fig S1. RMN ^{1}H and ^{13}C spectra for compound C2 in DMSO-d_6.

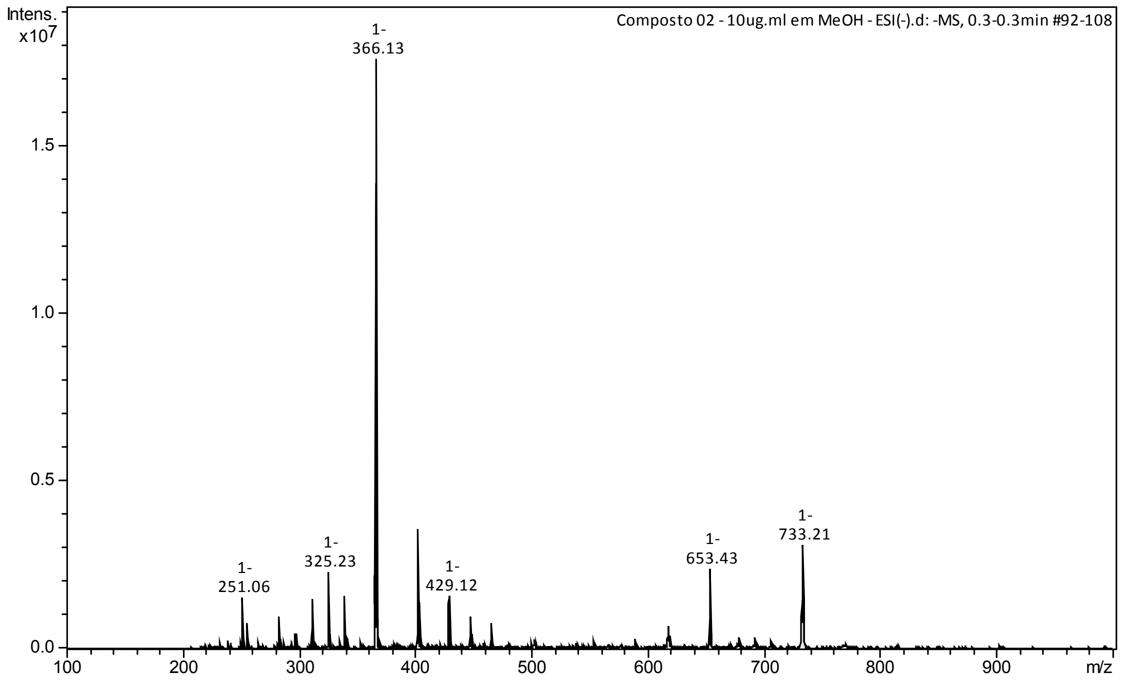

Fig S2. MS spectrum for compound C2.

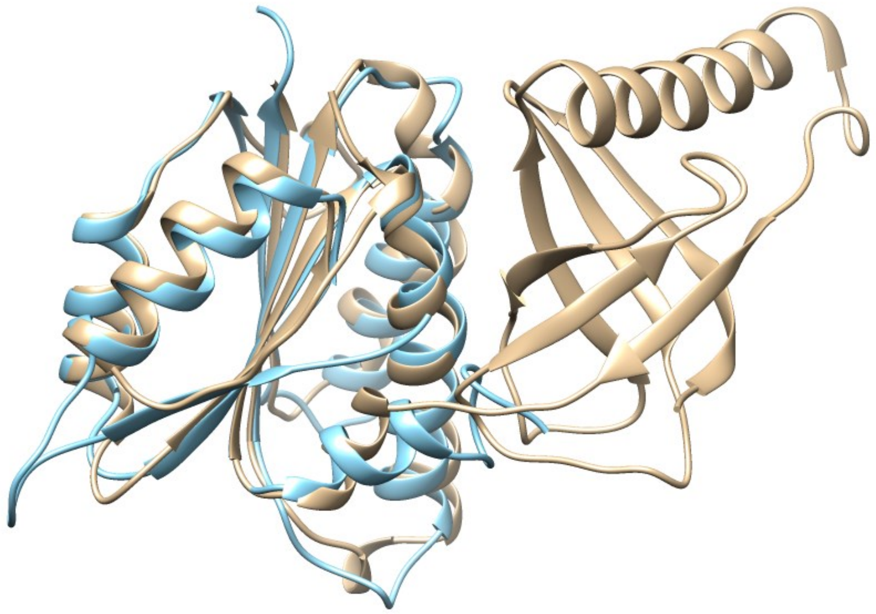

Fig S3. Structural superposition of NOX2 (PDB 3A1F – blue) and NOX5 (PDB 5O0X – brown). The study was performed using the UCSF Chimera software (RMSD 1.518). The FAD-binding domain is absent in NOX2.
